# Correlates of sugar-sweetened beverage consumption of Malaysian preschoolers aged 3 to 6 years

**DOI:** 10.1186/s12889-020-08461-7

**Published:** 2020-04-25

**Authors:** Leng Huat Foo, Ying Huoy Lee, Che Yahya Suhaida, Andrew P. Hills

**Affiliations:** 1 Programme of Nutrition and Dietetics, School of Health Sciences, Universiti Sains Malaysia, Health Campus, 16150 Kubang Kerian, Kelantan Malaysia; 2 School of Health Sciences, College of Health and Medicine, University of Tasmania, Newnham, Launceston, TAS 7250 Australia

**Keywords:** Sugar-sweetened beverage (SSB), Dietary factors, Physical activity (PA), Screen-based sedentary practice, Preschoolers

## Abstract

**Background:**

There is little information about the diet, lifestyle and parental characteristics associated with habitual sugar-sweetened beverage (SSB) consumption in Asian children. The aim of the present study was to assess cross-sectional associations between habitual SSB consumption and preschoolers’ diet, physical activity, sedentary behaviour as well as parental and child characteristics in Malaysian preschoolers aged 3 to 6 y.

**Methods:**

A total of 590 preschoolers, comprising 317 boys and 273 girls were included. Pre-pilot parental questionnaires were used to assess diet, physical activity (PA) and sedentary behaviour practices and anthropometry was assessed in preschoolers and their parents.

**Results:**

Multiple logistic regression analyses showed that preschoolers with more frequent weekly intake of snacks [OR 2.7; 95% CI, 1.6–4.4; *p* < 0.001] and monthly fast food consumption [OR 3.5; 95% CI, 1.9–6.3; *p* < 0.001], were associated with higher SSB intake (≥5 days in a week), after adjustments of potential confounders. Preschoolers with higher daily fruit and vegetable intake had lower SSB intake [OR 0.4; 95% CI, 0.2–0.8; *p =* 0.011]. A positive association of higher weekly vigorous PA [OR 2.0; 95% CI, 1.1–3.7; *p* = 0.030] and daily screen-based practices [OR 2.0; 95% CI, 1.2–3.6; *p* < 0.001] on habitual SSBs intake was also substantiated.

**Conclusion:**

Multiple diet, physical activity and sedentary behaviour factors were significantly associated with SSB intake among Malaysian preschoolers. Continued effort is required to encourage healthier beverage choices, as well as healthy diet and active lifestyle practices among children during the critical early years of growth and development.

## Background

Over recent decades, the prevalence of childhood obesity has increased more rapidly in low- and middle-income countries than in developed countries [1]. The worldwide prevalence of obesity among children aged 5 years and younger has risen from 4.2% in 1990 to about 6.7% in 2010 with a projected increase to 9.1% by 2020 [2]. Rapid increases in the prevalence of childhood obesity is of particular concern because of the enhanced risk of adverse health outcomes during childhood and later in life, including cardiovascular disease, type-2 diabetes, and various cancers [1]. In Malaysia, most studies have focused on older children and adolescents rather than preschoolers with only two published studies on this population [3, 4]. Prevalence of obesity in these studies, as determined by BMI-for-age, was between 8.1 and 9.3%, suggesting that childhood obesity is of critical concern for the youngest children in Malaysia [3, 4].

There is an urgent need for a comprehensive understanding of the potential diet and lifestyle determinants of excess weight gain and risk of obesity in preschoolers. The preschool years are a critical period of rapid growth and development with implications for life-long health status [5, 6]. For instance, dietary patterns in children include consumption of more high energy-dense foods, high in fat, sugar and salt [7–9]. At the same time, lower physical activity and more sedentary lifestyle practices are evidenced [7–9]. A rapid increase in high-calorie beverage consumption is increasingly recognized as a matter of concern, especially among young children, not only because such beverages provide a high caloric value with little or no nutritional value and also because they replace other healthy food choices [6, 10]. SSBs are a leading source of added sugar in our diet and consumption has increasingly become the drink of choice of younger children [11, 12]. Carbonated soda drinks, flavoured fruit drinks and milk-based beverages are being consumed in increasing amounts by young children and have become major sources of added sugar in their diet [13]. A growing body of evidence suggests that high consumption of SSBs is significantly associated with low intakes of micronutrients [14, 15], increased risk of dental caries [16], and risk of excess weight gain, and metabolic disorders in children [10, 17–19]. Moreover, numerous studies have also shown that dietary practices established in early childhood could persist into adolescence [20], and then into adulthood [21]. Therefore, to address the risk of excessive weight gain and other adverse health effects in children it is necessary to identify factors associated with a high intake of SSB among young children. If we can identify the factors that influence the high consumption of SSB early in life, strategies could be developed to prevent the development of unhealthy eating habits and reduce the risk of cardiometabolic disorders later in life.

Research to date has attempted to identify the influence of diet, physical activity and sedentary-related factors of children as well as parental socio-demographic characteristics on SSB consumption, but research has focused mainly on older children and adolescents [11, 17, 20]. Very few studies have been conducted in children of preschool age, including Malaysia [19, 22]. Garnett and colleagues [22] identified that about half of the 2 year-old children studied consumed SSBs at least once a week, and household income and ethnicity were significantly associated with habitual SSB intake. Another study of Australian children aged 2 to 16 years reported that age and socio-economic status (SES) were significantly associated with habitual SSB intake, high intakes were found in older children and in children from lower SES [23]. In these studies, only limited information was obtained about diet, SES and physical activity [22, 23]. Other factors such as sedentary behaviors and parental characteristics were not examined, making it difficult to determine the significance of multifactorial predictors on SSB consumption patterns in young children. To the best of our knowledge, no study of SSB consumption and its determinants has been conducted among children from diverse ethnic backgrounds in Asia. A comprehensive understanding of habitual SSB intake and associated factors in preschoolers would help to develop strategies to improve the healthy nutrition practices during the early years of growth and development. We hypothesized that both child diet, physical activity and sedentary practices and parental characteristics are significantly related to children’s habitual SSB consumption. Therefore, the main aim of the present study was to assess associations between habitual SSB consumption and preschoolers’ diet, physical activity, sedentary practices, as well as child (age, gender and ethnicity) and parental (weight status, educational, household size and income status) characteristics among Malay and Chinese preschoolers aged 3 and 6 y in Malaysia.

## Methods

### Study design

Preschoolers aged 3 to 6 y (317 boys and 273 girls) were recruited from urban and suburban areas of the district of Kota Bharu, Kelantan, Malaysia. Children were recruited from 16 preschool centers, randomly selected from 169 kindergartens registered with the State Education Department of Kelantan, Malaysia. Of these, about 8 kindergartens were randomly selected for Malay preschoolers; whereas 8 kindergartens from a total of 13, with at least 50% Chinese children, were randomly selected to recruit Chinese children. Parents or guardians of children selected were invited to participate in the study. Detailed explanations and discussions were conducted with the person-in-charge of selected preschool centers such as the principal and/or teachers, and parents or guardians. This included the nature, purpose, and the detailed measurement procedures used in the study and data collection was carried-out during the school day. Children were eligible to participate in this study if they met the following inclusion criteria: 3 to 6 years, being healthy and physically active i.e., free from any physical or mental disability, and not taking any long-term medication associated with any medical health problems that could influence their nutritional and health status. The two different measurements, a self-administered parental questionnaire and anthropometric measurements of the children were carried-out by researchers either at school or in the home. The questionnaire was completed by a parent (94.9%), mainly by the mother (*n* = 539). Each completed parental questionnaire was checked by trained nutrition students for completeness and accuracy. The present study was approved by the Human Research Ethics Committee of the Universiti Sains Malaysia (USM). Written informed consent was obtained from either the parent or guardian of the children prior to the study.

### Anthropometry assessments

Anthropometric measurements were conducted by the researcher according to a standardized World Health Organization (WHO) procedure [23]. In brief, each child was weighed (wearing light clothing and without shoes) to the nearest 0.5 kg with a mechanical weighing scale (Model: Seca 762, Germany). Height of child with bare feet was measured using a stadiometer (Model: Seca 206, Germany), to the nearest 0.1 cm. Body mass index (BMI) was calculated as body weight in kilograms divided by height in meters squared (kg/m^2^). Waist and hip circumference were measured with an inelastic measuring tape. All anthropometric measurements were taken twice and an average value was calculated. For the parental anthropometry assessment, BMI of parents were calculated based on their self-reported height and weight and categorized in accordance with the WHO standard for adults [23].

### Child and parental socio-demographic factors and child diet, physical activity and sedentary behaviour

A self-administered parental questionnaire was used to profile socio-demographic and economic status of both parents as well as diet, physical activity and sedentary practices of children. The questionnaire was formulated based on a pilot study involving 50 parents who had children of less than 6 years of age in Kelantan. Detailed information on diet, physical activity and sedentary behaviours of pre-schoolers, both at home and at preschool centers, was also obtained. Information on frequency, serving size, and common types of foods consumed for breakfast, and snacks during the previous week were gathered. Fast food consumption such as type and frequency of intake over the previous month were assessed. Snack time refers to any food and drinks taken between the three main meals in a day. In addition, frequency and common types of fruits, vegetables, all beverage consumption, including SSBs such as type and frequency of intake over the past week were obtained. Detailed information on type, frequency and duration of sport-related activity was also gathered. Vigorous physical activity was determined based on any leisure activity or organized sports played by children at least 60 min in the past week that made them sweat and feel “out of breath”. Detailed information on frequency and duration of activities involving electronic devices (television viewing, video games and computer and/or internet use), both on weekdays and on weekends was also collected.

### Classification of sugar-sweetened beverages

SSB intake was defined as any non-diet, non-alcoholic beverages with added sugars including brown sugar, cane sugar, granulated sugar, dextrose, honey, and any types of syrups such as high-fructose corn syrup and table syrup [24]. However, added sugars did not include naturally occurring sugars such as lactose in milk and fructose in fruits [24]. Fruit juice (100%), fresh fruit juice with less than 2% added sugars and milk were excluded from the SSB category. Four types of SSB are commonly consumed in Malaysia, namely i). coffee, tea or rose syrup with added sugar or condensed milk, sweetened packaged drinks (ready-to-drink packaged beverages containing white sugar or cane sugar) and sweetened malted drinks with primary ingredients such as skimmed milk powder, malto-dextrin, and sugar (equivalent to approximately 18 to 40 g added sugars per 250 mL serving) [25]; ii). sugar-sweetened soda beverages comprising all types of soda, pop, sport drinks, and fizzy drinks containing about 35 to 42 g of sugar per 355 mL (~ 1 can of soda beverage); iii). fruit drinks that include all concentrated fruit juices (25–50% juice from fruit) with sweetener and fruit-flavored drinks such as squash, juice drink, and juice cordial with 10–20% fruit juice (equivalent to approximately 24–38 g added sugar per 250 mL serving), and iv). flavored milk-based drinks including commercially available cultured and fermented milk products with approximately 15–19 g of sugar in 100–200 mL.

### Statistical analysis

Data were analyzed using the SPSS software version 20.0 for Windows (SPSS Inc., Chicago, IL, USA). A gender- and age-specific z-score of BMI-for-age was calculated using AnthroPlus version 1.0.4 [26]. BMI classifications were based on gender- and age-specific cut-off points recommended by the WHO [27]. BMI z-score of less than − 2 SD, − 2 ≤ SD ≤ 1 SD and > + 1 SD were classified as thinness, normal weight and, overweight and obese, respectively. For the analysis of the exposure variables, firstly, household income of RM3000 is used as a reference because this amount is commonly and widely used by the government to distinguish low and high household earning power [﻿28﻿]. Secondly, the educational status of parents was assessed from the highest level of education attained by both parents. In Malaysia, compulsory school attendance of parents included in this study based on their age was 12 years (mean age of father and mother was 34.2 and 32.2 years, respectively). Therefore, we decided to use two categories: “never having been to secondary school” and “up to tertiary education”. Tertiary education is referred to as post-secondary education, ranging from vocational education to a university degree.

All variables were tested for normality using the Kolmogorov-Smirnov test prior to any statistical comparisons. Descriptive statistics were reported as mean values and standard deviations, unless otherwise indicated. An independent *t*-test and *Pearson* chi-square test were used to determine between-gender differences on all variables. Simple and multiple logistic regression analyses were used to examine the odds ratios of high habitual SSB intake, as defined by ≥5 days a week, including the following exposure variables in the models; breakfast intake, fast food, snacks, fruit and vegetables consumption, weekly vigorous physical activity status and daily screen-based levels and parental socio-economic and BMI status exposure variables taking into account potential confounders such as age, gender, ethnicity, household income, number of siblings, parental socio-demographic, economic and BMI status, dietary practices, physical activity and screen-based sedentary duration in this multivariate analysis. Furthermore, interactions between diet, physical activity and screen-based sedentary practice of children were also tested but no significant interactions were found. A statistical significance for all tests was defined by *p* value of < 0.05.

## Results

A total of 590 Malay and Chinese preschoolers aged 3 to 6 y, comprising 317 boys and 273 girls were included in the study. Table 1 shows the general characteristics, diet, physical activity and sedentary behaviour of the preschoolers. The mean age of the participants was 4.6 ± 0.9 years and mean BMI 14.3 ± 1.8 kg/m^2^. The majority (76.4%) were normal weight according to the new revised WHO Child Growth Standard reference criteria [﻿28﻿] and the prevalence of thinness (20.7%) was higher compared to the overweight and obese group (2.9%). In general, boys had significantly higher body weight (*p* = 0.019) and height (*p* < 0.001) compared to girls. Preschool boys had significantly higher weekly breakfast consumption (*p* < 0.001), and lower frequency of weekly snack intake (*p* < 0.001) compared to girls, whereas a higher proportion of girls had higher monthly fast food intake (*p* = 0.011), and fruit and vegetable intake (at least 2 servings a day) (*p* < 0.001). In contrast, boys spent more time in daily screen-based sedentary practices (*p* = 0.007). For the parental socio-demographic and weight status, maternal BMI levels were higher for girls than boys (*p* = 0.003), whereas maternal educational status was higher in boys compared to girls (*p* = 0.027). In addition, household size was significantly higher in boys’ families (*p* < 0.001). Habitual SSB consumption for the present study was expressed by frequency and common types of SSB per week. Children consumed on average, 5.0 ± 3.9 SSB per week, with more than half (61.2%) were consumed in weekly basis. Most common SSBs were sugar-added drinks such as coffee, tea, and flavored and malted beverages (46.0%), followed by sugar-sweetened soda drinks (22.7%), fruit-flavored drinks (15.8%), and flavored milk drinks (15.5%). In general, a similar pattern of SSB consumption was found for boys and girls.

**Table 1:** Descriptive characteristics of a cohort of 590 Malaysian preschoolers participating in a study on sugar-sweetened beverages

	Boys (*N* = 317)	Girls (*N* = 273)	Total (*N* = 590)	*P* value
	Mean ± SD			
Race				
-Malay (%)	65.3 (207)	63.0 (172)	64.2 (379)	0.562
-Chinese (%)	34.7 (110)	37.0 (101)	35.8 (211)	
Age (years)	4.7 ± 0.8	4.4 ± 0.9	4.6 ± 0.9	0.001
Body weight (kg)	15.3 ± 3.5	14.6 ± 3.7	15.0 ± 3.6	0.019
Height (m)	1.0 ± 0.1	1.0 ± 0.1	1.0 ± 0.1	< 0.001
BMI (kg/m^2^)	14.3 ± 1.8	14.2 ± 1.8	14.3 ± 1.8	0.657
BMI z-score^a^	−0.9 ± 1.2	−1.1 ± 1.4	− 1.0 ± 1.3	0.062
-Thinness	12.9 (41)	29.7 (81)	20.7 (122)	< 0.001
-Normal weight	83.9 (266)	67.8 (185)	76.4 (451)	
-Overweight and obese	3.2 (10)	2.6 (7)	2.9 (17)	
Waist circumference (cm)	49.9 ± 4.8	50.4 ± 4.9	50.1 ± 4.8	0.200
Hip circumference (cm)	55.4 ± 4.7	56.0 ± 5.7	55.7 ± 5.2	0.152
Weekly breakfast status				
- < 5 times/week	44.8 (142)	62.6 (171)	53.1 (313)	< 0.0001
- ≥5 times/week	55.2 (175)	37.4 (102)	46.9 (277)	
Monthly fast food status				
- Yes	58.4 (185)	68.5 (187)	63.1 (372)	0.011
Weekly snack status				
- < 5 times/week	64.5 (182)	43.0 (105)	54.6 (287)	< 0.0001
- ≥5 times/week	35.5 (100)	57.0 (139)	45.4 (239)	
Daily fruit and vegetable status				
- < 2 servings/day - ≥2 servings/day	93.1 (295) 6.9 (22)	83.5 (228) 16.5 (45)	88.6 (523) 11.4 (67)	< 0.0001
Weekly vigorous PA level status				
- Yes	68.1 (216)	68.5 (187)	68.3 (403)	0.925
Daily screen-based sedentary practice				
- < 2 h/day	48.6 (154)	59.7 (163)	53.7 (317)	0.007
- ≥2 h/day	51.4 (163)	40.3 (110)	46.3 (273)	
SSB consumption status per week				
- Yes (%)	59.9 (190)	62.6 (171)	61.2 (361)	0.279
Frequency (times/week)	5.0 ± 4.4	4.8 ± 3.4	5.0 ± 3.9	0.630
Types of SSB^b^				
- Sugar-added beverages	44.2 (84)	48.0 (82)	46.0 (166)	
- Carbonated soda beverages	26.3 (50)	18.7 (32)	22.7 (82)	
- Fruit-flavoured drinks	16.3 (31)	15.2 (26)	15.8 (57)	
- Flavoured milk drinks	13.2 (25)	18.1 (31)	15.5 (56)	
***Parental characteristics***				
Household size	5.7 ± 1.7	5.1 ± 1.5	5.4 ± 1.6	< 0.001
Number of siblings	2.9 ± 1.5	2.7 ± 1.3	2.8 ± 1.4	0.075
Household income (RM/month)				
- <RM3000.00	42.4 (134)	45.8 (125)	44.0 (259)	0.410
- ≥RM3000.00	57.6 (182)	54.2 (148)	56.0 (330)	
***Father***				
Age (years)	34.4 ± 7.1	34.0 ± 7.4	34.2 ± 7.2	0.515
BMI (kg/m^2^)^c^	25.8 ± 3.9	26.2 ± 3.1	26.0 ± 3.6	0.218
- Underweight	1.4 (4)	0.7 (2)	1.1 (6)	< 0.0001
- Normal weight	47.4 (135)	33.2 (89)	40.5 (224)	
- Overweight	36.8 (105)	59.0 (158)	47.6 (263)	
- Obese	14.4 (41)	7.1 (19)	10.8 (60)	
Paternal educational status				
- Never to secondary school	51.4 (163)	56.4 (154)	53.7 (317)	0.233
- Up to tertiary education	48.6 (154)	43.6 (119)	46.3 (273)	
***Mother***				
Age (years)	32.4 ± 6.0	31.9 ± 6.1	32.2 ± 6.0	0.257
BMI (kg/m^2^)^c^	23.0 ± 5.6	24.3 ± 4.7	23.6 ± 5.2	0.003
- Underweight	14.5 (46)	7.7 (21)	11.4 (67)	0.002
- Normal weight	61.8 (196)	56.0 (153)	59.2 (349)	
- Overweight	15.5 (49)	24.9 (68)	19.8 (117)	
- Obese	8.2 (26)	11.4 (31)	9.7 (57)	
Maternal educational status				
- Never to secondary school	46.4 (147)	55.7 (152)	50.7 (299)	0.027
- Up to tertiary education	53.6 (170)	44.3 (121)	49.3 (291)	

Abbreviations: *BMI* body mass index; *PA* physical activity; *SSB* Sugar-sweetened beverage

^a^Classification of BMI-for-age based on the recommended cut-off point of new revised WHO growth reference (WHO, 2008)

^b^Sugar-added beverages included all conventional beverages such as coffee, tea, rose syrup-flavored, packaged, and malted beverages; carbonated soda beverages included soda, pop, coke, sport drink, and fizzy drink; fruit-flavored drinks included concentrated fruit juices and fruit-flavored beverages and flavored milk drinks included cultured and fermented milk-based products (See details of the classification of SSBs in method section)

^c^Classification of BMI (WHO, 2005)

Significant difference between boys and girls at ^*^p < 0.05, ^**^p < 0.01, and ^***^p < 0.001

Table 2 shows odds ratios (OR) for associations between child habitual SSB consumption and child diet, physical activity and sedentary practices, as well as child and parental socio-demographic characteristics and parental weight status on habitual SSB consumption. In the unadjusted logistic regression models, gender, ethnicity, number of siblings, household income, and paternal education status, dietary practices of breakfast, snack intake, fast food consumption, and daily fruit and vegetable intake, lifestyle-related factors such as weekly vigorous PA status and daily screen-based sedentary practice were significantly associated with the habitual SSB intake. However, when all variables were included in a multivariate logistics regression model, only some remained statistically significant. Children with 4 siblings or more had significantly higher weekly consumption of SSBs [OR 3.08; 95% CI: 1.80–5.26; *p* < 0.001] than those with three or fewer siblings. For dietary practices, children with higher snack intakes of 5 times or more in a week [OR 2.88; 95% CI, 1.72–4.84; *p* < 0.001] and monthly intake of fast food of at least once per week [OR 3.76; 95% CI, 2.04–6.92; *p* < 0.001] had significantly higher odds of SSB intake compared to those with lower intakes of snack and fast food groups, respectively. On the contrary, preschoolers with higher fruit and vegetable intake (2 servings or more in a day) had significantly lower intake of SSBs [OR 0.41; 95% CI, 0.21–0.81; *p =* 0.011] compared to those with lower daily intake. Children who either reported weekly participation in vigorous PA [OR 1.99; 95% CI, 1.07–3.69; *p* = 0.030] or 2 h or more of daily screen time [OR 1.99; 95% CI, 1.13–3.51; *p* < 0.001], had significantly higher weekly intake of SSBs, after full adjustment of all potential confounders.

**Table 2:** Crude- and adjusted logistic regression analysis of associations between SSB consumption of preschoolers and diet, physical activity and socio-demographic factors^a^ (*N* = 590)

	Odds ratio [95% CI]			
	Crude	***p***-value	Adjusted	***p***-value^**a**^
High SSB consumption (≥5 days/ week)	28.5% (168)			
Age				
- 3 to 4 years	1.00 [referent]		1.00 [referent]	
- 5 to 6 years	0.86 (0.57–1.30)	0.485	1.17 (0.67–2.03)	0.584
Gender				
- Boys	1.00 [referent]		1.00 [referent]	
- Girls	1.67 (1.16–2.39)	0.005	1.30 (0.78–2.16)	0.319
Race				
- Malay	1.00 [referent]		1.00 [referent]	
- Chinese	2.32 (1.61–3.35)	< 0.0001	1.79 (0.83–3.85)	0.107
Number of siblings				
- 0 to 3	1.00 [referent]		1.00 [referent]	
- ≥4 siblings	2.00 (1.39–2.89)	< 0.0001	3.08 (1.80–5.26)	< 0.001
Household income				
- <RM3,000	1.00 [referent]		1.00 [referent]	
- ≥RM3,000	1.73 (1.20–2.51)	0.004	1.20 (0.89–2.83)	0.120
Paternal BMI status				
- Underweight	1.00 [referent]		1.00 [referent]	
- Normal weight	0.28 (0.06–1.43)	0.126	0.60 (0.06–5.74)	0.659
- Overweight	0.62 (0.12–3.15)	0.567	2.16 (0.22–21.34)	0.509
- Obese	0.22 (0.04–1.27)	0.090	0.12 (0.01–1.41)	0.093
Paternal educational status				
- Never to secondary school	1.00 [referent]		1.00 [referent]	
- Up to tertiary education	0.65 (0.45–0.94)	0.020	0.71 (0.35–1.42)	0.332
Maternal BMI status				
- Underweight	1.00 [referent]		1.00 [referent]	
- Normal weight	0.65 (0.37–1.16)	0.143	0.28 (0.12–0.64)	0.103
- Overweight	1.69 (0.90–3.19)	0.103	0.422 (0.17–1.07)	0.069
- Obese	0.86 (0.39–1.86)	0.691	0.539 (0.19–1.55)	0.253
Maternal educational status				
- Never to secondary school	1.00 [referent]		1.00 [referent]	
- Up to tertiary education	0.94 (0.66–1.35)	0.754	0.93 (0.49–1.75)	0.811
Dietary and lifestyle-related practices				
Weekly breakfast consumption				
- < 5 times/ week	1.00 [referent]		1.00 [referent]	
- ≥5 times/ week	1.66 (1.16–2.38)	0.006	1.88 (0.86–4.09)	0.112
Weekly snack intake				
- < 5 times/ week	1.00 [referent]		1.00 [referent]	
- ≥5 times/ week	2.43 (1.65–3.58)	< 0.0001	2.88 (1.72–4.84)	< 0.001
Monthly fast food intake				
- No	1.00 [referent]		1.00 [referent]	
- Yes	4.82 (3.03–7.68)	< 0.0001	3.76 (2.04–6.92)	< 0.001
Daily fruit and vegetable intake				
- < 2 serving/day	1.00 [referent]		1.00 [referent]	
- ≥2 servings/day	0.36 (0.21–0.60)	< 0.0001	0.409 (0.21–0.81)	0.011
Weekly vigorous physical activity status				
- No	1.00 [referent]		1.00 [referent]	
- Yes	2.24 (1.47–3.43)	< 0.0001	1.99 (1.07–3.69)	0.030
Daily screen-based sedentary practice				
- < 2 h/day	1.00 [referent]		1.00 [referent]	
- ≥2 h/day	1.83 (1.27–2.65)	0.001	1.99 (1.13–3.51)	< 0.001

^a^Adjusting for age, gender, ethnicity, household income, number of siblings, child dietary practices, physical activity and daily screen-based sedentary practice and the parental demographic and socio-economic characteristics, and parental BMI

## Discussion

The main findings of the study indicate that high intakes of fast food and snacks, low intake of fruit and vegetables, high weekly vigorous PA, and high daily use of screen-based media as well as having more than three siblings were significantly associated with more frequent intake of SSBs. This was after full adjustment for age, gender, ethnicity, BMI, parental socio-demographic and economic status, diet, physical activity and screen-based sedentary practice confounders. This result suggests that multiple factors are associated with SSB intake in this group of preschoolers. To the best of our knowledge, this is the first study to document the diet, physical activity and sedentary practices of preschoolers, as well as parental weight status and socio-economic characteristics on SSB intake. Other studies have been more limited in focus and assessed a limited range of nutrients or foods and socio-demographic status [22, 23, 29], and parental perception on children’s SSB intake [12]. Not surprisingly, as parents have the greatest influence on their children’s eating habits, it is important to identify child and parent-related factors associated with SSB intake. Although significant associations were found between a child’s sex and ethnicity and parental BMI and SES status, and SSB intakes, these differences were diminished after adjustment for other potential confounders. Only diet, physical activity and screen-based sedentary behaviours remained statistically significant after adjustment suggesting that multiple factors might influence SSB consumption during the early years of growth and development.

Fast food and snack consumption were significantly associated with greater consumption of SSB, similar to findings of several studies conducted in Western countries [30, 31]. This relationship might be explained by SSBs such as sodas, colas, and fruit drinks, often being consumed as part of a typical fast food meal. Similarly, higher snack consumption was associated with high habitual SSB intake, a finding consistent with Saudi children and adolescents aged 10–19 years. SSB consumption was positively associated with high savory and sweet snack intakes [17]. In contrast, a significant negative association was found between high intakes of fruit and vegetable, and SSB consumption in these preschoolers in line with numerous other studies in the US [﻿19﻿], China [﻿20﻿] and Greece [32]. This suggests that SSB consumption is related closely to the overall quality of children’s diet. A study of U.S. children aged between 2 to 11 years reported inverse associations between the consumption of soda and sweetened beverage intakes and fruit and vegetable intakes [33]. Similarly, a study of adolescents with a mean age of 15 years also reported that consumption of soda beverages was associated with lower intakes of healthy foods including fruit and vegetables [34]. Surprisingly, children in our study with higher weekly vigorous PA status had significantly higher intakes of SSB compared to those with less vigorous levels of PA, a finding contrary to that of a study conducted among US adolescents aged 14 to 18 y [﻿35﻿]. Nevertheless, the present findings are partly consistent with another study of adolescents, in which higher physical activity was associated with higher intakes of flavored and sports beverages [﻿34﻿]. Although there is no clear explanation for a positive association between SSB intake and vigorous PA among these preschoolers in the present study, several hypotheses come to mind. Children with higher PA levels tend to consume more beverages, including SSBs, as part of the fluid intake needed to maintain hydration, especially when engaging in vigorous PA in high temperatures and humidity common in Malaysia. Therefore, when children engage in vigorous PA there should be continued efforts to encourage healthier drink choices such as water or drinks low in sugar. The sedentary practice of television watching and computer use for 2 h or more was significantly associated with higher SSB intakes. This is similar to reports of US adolescents aged 14 to 18 years, in which higher consumption of SSB was associated with a greater amount of time spent watching television or playing video or computer games for 2 h or more per day [﻿35﻿]. Another possible explanation for the high consumption of SSB associated with sedentary computer or television practice could be the increased exposure to mass media advertising for SSBs [36]. Another unexpected finding was the positive significant association between the number of siblings and SSB intake after adjusting for other confounding variables. This finding was partly consistent with those of a nationwide study of US households, which indicated that household size was significantly and positively associated with SSBs [37]. These findings suggest that family size might affect children’s SSB intake. In contrast, no such association was found between household size and carbonated soft drinks [38]. Although the precise mechanism to explain this positive links between family size, as expressed by numbers of siblings and SSB intake is still unknown, a speculative explanation may be that socio-economic disadvantage a proxy indicator associated with higher numbers of children in household [39]. Thus, children with low socioeconomic status tend to consume more SSBs in relation to their overall diet than children from higher income groups [22, 40].

Based on the current pattern of SSB consumption of preschoolers, conventional sugar-added beverages such as coffee, tea, malted and other conventional flavored beverages were still very popular, however, the commercial availability of ready-to-drink SSBs is increasing. Almost 54% of children reported consuming SSBs each week, a consumption pattern consistent with reports from other countries. For example, non-traditional sugar-added beverages such as fruit drinks and sports/energy-related beverages are becoming a common choice compared to soda beverages among children aged 2 to 11 years in the United States [11]. Other studies also supported the increasing consumption of SSBs such as fruit drinks, fruit juice, and squashes other than soda beverages among preschoolers in Canada [41], Australia [42] and Jordan [43]. This suggests the necessity to reconsider public health policies to reduce SSB consumption.

Significant gender differences on BMI, household size, measures of eating practices, and sedentary practices, and parental BMI and educational status were also found. For instance, the percentage of girls who were underweight is very high at 29.7%, twice that of boys (12.9%). In addition, girls were less likely to consume breakfast at least 5 days a week, and were more likely to eat snacks. This raises nutritional concerns about the possible underfeeding of young girls and the preferential feeding of young boys. Although the precise mechanisms are not understood, it is generally agreed that underweight in children is significantly associated with socioeconomic hardship at the household level. In the present study, there was a significant inverse association between maternal education status and a child’s BMI (*p* < 0.0001), whereas no such significance was found between maternal BMI and a child’s BMI (data not shown). This finding suggested that higher levels of maternal education were significantly associated with lower risk of being underweight. Moreover, maternal educational status of preschool boys was significantly higher compared to girls, which could possibly explain the relatively high number of girls who were underweight. Therefore, the present finding emphasises the need for nutritional and health promotion intervention programs of younger children tailored to families with a low level of education.

### Limitations and strengths of the study

The present study has some limitations. First, it was cross-sectional in design; therefore a causal relationship between factors studied and SSB intake cannot be established. Secondly, we had no information on the exact quantity of SSBs consumed by the children in the study, therefore detailed information was obtained on the common pattern (types and frequency) of consumption of SSBs from various places such as at home, nursery or preschool centers. Thirdly, parental weight and height were self-reported and this may have resulted in some misclassification of overweight and obesity. Fourthly, it is generally agreed that any epidemiological research involving recall is prone to recall bias or errors caused by systematic differences in the accuracy or completeness of recollections by study parents regarding events or experiences from the past. In the present study, recall bias was minimised because of the relatively short recall duration of the past week. Recalling the dietary habits and lifestyle practices across the previous week was a relatively low burden to the parents or guardians and provided an accurate assessment of the pattern of food consumption and lifestyle practice of children. Such an approach takes into account day-to-day variation attributed to interactions between biologic and environmental influences such as appetite, weekday, weekends, physical activity, leisure time, and personal economic conditions and is consistent with previous studies among young children [44, 45] and children and adolescents [46]. Lastly, because the study was limited to Malay and Chinese preschoolers living in Malaysia, the present findings may not be consistent for other populations. On the other hand, the strength of this study should be acknowledged including the large sample size. Comprehensive body composition data and other important factors such as parental characteristics, diet, physical activity and sedentary-related factors were included in the multivariate analysis model. The exploration of various interacting factors is a further strength of this study.

## Conclusions

A large proportion of preschool-aged children in Malaysia consume SSBs every week with consumption significantly associated with intake of fast food, snacks, low fruit and vegetable intake, vigorous physical activity, sedentary practices, and number of siblings after adjusting for potential confounding factors. Based on the current findings, ongoing efforts are needed to provide healthy eating environments, both at home and at preschool centers. This would include the provision of a variety of fruit and/or vegetables as snack intake for preschoolers in order to decrease the intake of SSB, with the consequent benefit of preventing adverse health effects such as excessive weight gain, and the risk of obesity and cardiometabolic disorders during childhood and in later life. Further research is needed to confirm these findings in a large population and to explore possible dietary and lifestyle intervention strategies to reduce the habitual consumption of SSB among growing children during the preschool years.

## Data Availability

The datasets used and/or analysed during the current study are available from the corresponding author on reasonable request.

## Abbreviations

SSB: Sugar-sweetened beverage
PA: Physical activity
OR: Odds ratios
SES: Socio-economic status
WHO: World Health Organization
BMI: Body mass index
